# Combining Awake Anesthesia with Minimal Invasive Surgery Optimizes Intraoperative Surgical Spinal Cord Stimulation Lead Placement

**DOI:** 10.3390/jcm11195575

**Published:** 2022-09-22

**Authors:** Philippe Rigoard, Amine Ounajim, Lisa Goudman, Chantal Wood, Manuel Roulaud, Philippe Page, Bertille Lorgeoux, Sandrine Baron, Kevin Nivole, Mathilde Many, Emmanuel Cuny, Jimmy Voirin, Denys Fontaine, Sylvie Raoul, Patrick Mertens, Philippe Peruzzi, François Caire, Nadia Buisset, Romain David, Maarten Moens, Maxime Billot

**Affiliations:** 1 PRISMATICS Lab (Predictive Research in Spine/Neuromodulation Management and Thoracic Innovation/Cardiac Surgery), Poitiers University Hospital, 86021 Poitiers, France; 2 Department of Neuro-Spine & Neuromodulation, Poitiers University Hospital, 86000 Poitiers, France; 3 Pprime Institute UPR 3346, CNRS, ISAE-ENSMA, University of Poitiers, 86000 Poitiers, France; 4 Department of Neurosurgery, Universitair Ziekenhuis Brussel, Laarbeeklaan 101, 1090 Brussels, Belgium; 5 STIMULUS Consortium (reSearch and TeachIng neuroModULation Uz bruSsel), Vrije Universiteit Brussel, Laarbeeklaan 103, 1090 Brussels, Belgium; 6 Research Foundation—Flanders (FWO), 1090 Brussels, Belgium; 7 Department of Neurosurgery, Bordeaux University Hospital, 33000 Bordeaux, France; 8 Department of Neurosurgery, Colmar Hospital, 68000 Colmar, France; 9 Centre Hospitalier Universitaire de Nice, Department of Neurosurgery, Université Côte d’Azur, 06000 Nice, France; 10 FHU InovPain, Côte Azur University, 06000 Nice, France; 11 Department of Neurosurgery, Nantes University Hospital, 44000 Nantes, France; 12 Department of Neurosurgery, Lyon University Hospital, 69000 Lyon, France; 13 Department of Neurosurgery, Reims University Hospital, 51100 Reims, France; 14 Department of Neurosurgery, Limoges University Hospital, 87000 Limoges, France; 15 Department of Neurosurgery, Lille University Hospital, 59000 Lille, France; 16 Physical and Rehabilitation Medicine Unit, Poitiers University Hospital, University of Poitiers, 86021 Poitiers, France; 17 Department of Radiology, Universitair Ziekenhuis Brussel, Laarbeeklaan 101, 1090 Brussels, Belgium

**Keywords:** PSPS, FBSS, SCS, surgical lead, SCS implantation, MAST (for minimal access spine technologies), TCIVA (for target controlled intra-venous anesthesia), composite score, pain mapping, neuropathic pain, chronic pain, quality of life, anesthesia, hypnosis

## Abstract

Spinal cord stimulation (SCS) is an effective and validated treatment to address chronic refractory neuropathic pain in persistent spinal pain syndrome-type 2 (PSPS-T2) patients. Surgical SCS lead placement is traditionally performed under general anesthesia due to its invasiveness. In parallel, recent works have suggested that awake anesthesia (AA), consisting of target controlled intra-venous anesthesia (TCIVA), could be an interesting tool to optimize lead anatomical placement using patient intra-operative feedback. We hypothesized that combining AA with minimal invasive surgery (MIS) could improve SCS outcomes. The goal of this study was to evaluate SCS lead performance (defined by the area of pain adequately covered by paraesthesia generated via SCS), using an intraoperative objective quantitative mapping tool, and secondarily, to assess pain relief, functional improvement and change in quality of life with a composite score. We analyzed data from a prospective multicenter study (ESTIMET) to compare the outcomes of 115 patients implanted with MIS under AA (MISAA group) or general anesthesia (MISGA group), or by laminectomy under general anesthesia (LGA group). All in all, awake surgery appears to show significantly better performance than general anesthesia in terms of patient pain coverage (65% vs. 34–62%), pain surface (50–76% vs. 50–61%) and pain intensity (65% vs. 35–40%), as well as improved secondary outcomes (quality of life, functional disability and depression). One step further, our results suggest that MISAA combined with intra-operative hypnosis could potentialize patient intraoperative cooperation and could be proposed as a personalized package offered to PSPS-T2 patients eligible for SCS implantation in highly dedicated neuromodulation centers.

## 1. Introduction

Chronic pain, defined as pain experienced for more than 3 months [1], leads to psychological and social impairments that dramatically alter quality of life [2,3,4,5]. When conventional pharmacological and physical therapy fails, spinal cord stimulation is recommended and is considered as a useful tool to manage chronic refractory pain [6,7,8,9,10,11], including persistent spinal pain syndrome after surgery (PSPS-T2) [12], when the neuropathic component is significant [13,14,15,16,17]. During the last decades, technological refinements have shown promising perspectives in two directions:

- SCS temporal resolution has been the subject of intensive research, making new waveforms available for the last generation of internal pulse generators (IPG). This modulates temporal resolution of the electrical signal in view of obtaining better pain relief, less discomfort and more personalized therapy by selecting or even combining several signals at the same time [18,19,20,21,22,23]. This also identifies new SCS mechanisms of action conveyed by different patterns from the classical gate control theory [24,25,26,27]. oudmanth, Foremanste 2016, Goudman et al. supériorité., Billot Mns sa pratique cliniquetifique afin de les synthétiser.
- SCS spatial resolution has been optimized through the multiplication of contacts at the surface of implanted leads, generating more precise and complex electrical fields, in view of enhancing spatial neural targeting [21,28].

Wishing to obtain optimal paresthesia coverage using conventional tonic SCS in order to achieve better pain relief, we have started to implant surgical leads under awake anesthesia (AA) so as to optimize spatial neural targeting with or without hypnosis [13,29] in a dedicated operating theatre [30,31]. We initially developed this approach using multicolumn surgical lead implantation for back and leg pain indications in PSPS-Type 2 patients. To minimize surgical trauma, we developed a new surgical approach requiring minimal invasive surgery (MIS), based on minimal access spinal technologies (MAST) [32,33]. MAST allowed us to perform SCS surgical implantation under target controlled intra-venous anesthesia (TCIVA), making it possible to assess the patient intraoperatively with high-fidelity despite surgical lead invasiveness. Intraoperative testing using quantitative measurements of pain surface, pain intensity, pain typology and paresthesia coverage was implemented through an interactive tactile interface specifically developed for this purpose [5,21,34,35,36]. The mapping tool and software (Neuro-Mapping Locator^TM^/NML) provide novel indices to instantaneously assess/compare lead performance (percentage of pain area covered by paresthesia generated via SCS) and lead selectivity (percentage of paresthesia adequately overlapping painful territories) defining an “R index”, with intraoperative objective data. The added value of intraoperative assessment in an awake condition, in order to optimize lead SCS placement, has yet to be determined. We hypothesized that combining AA with MIS could improve SCS outcomes.

We designed a national prospective multicenter study to evaluate the ability of multicolumn SCS to optimize back pain coverage and pain relief using complex multicolumn programming [8], and to compare lead placement optimization in PSPS-T2 patients implanted with surgical leads using a broad spectrum of surgical approaches (ESTIMET study). This study was performed in 12 French expert centers. In the vast majority of centers, patients were implanted using minimal invasive surgery under general anesthesia (MISGA). In some centers, patients were implanted using traditional laminectomy under general anesthesia (LGA). All patients operated on in the center X, were implanted using a combination of MIS with AA (MISAA), consisting of TCIVA + intraoperative hypnosis [29], allowing for intraoperative mapping assessment with NML^TM^ software.

In the present retrospective ancillary study, our specific goal was to evaluate whether all the above-mentioned conditions deployed to optimize intraoperative patient feedback would contribute to optimize lead placement, and thereby obtain significantly better pain coverage and/or clinical outcomes.

## 2. Materials and Methods

### 2.1. Objectives

Our primary objective was to analyze the impact of lead placement approach on SCS performance (paresthesia coverage) in paresthesia-based stimulation mode. To achieve this, we compared lead performance and lead selectivity of multicolumn SCS between the three groups of implanted patients: MISAA group, MISGA group and LGA group. Our secondary objectives were to compare pain relief (global, back and leg), functional improvement, psychological distress, improvement in quality of life and global health after 12 months of follow-up.

### 2.2. Study Design

Our study was a retrospective ancillary study, where data were collected during ESTIMET, which was a multicenter prospective randomized controlled trial, including 115 PSPS-T2 patients eligible for SCS and implanted with surgical multicolumn SCS lead, in 12 French centers with a 1-year follow-up [8,37]. The primary objective of this study was to compare 6-month outcomes between multicolumn programming SCS and monocolumn programming SCS. A total of 104 patients were permanently implanted after a successful trial. As part of the ESTIMET study, data collection was conducted according to the guidelines of the Declaration of Helsinki and the French Data Protection Authority (CNIL, MR-001), and approved by the Ethics Committee “CPP Ouest III” and the French Agency for the Safety of Health Products “ANSM” (number: 2011-A01695-36). The ClinicalTrials.gov Identifier is: NCT01628237. All subjects involved in the study provided written informed consent.

### 2.3. Study Population

The study population consisted of PSPS-T2 patients, defined as patients suffering from persistent back and leg pain for at least six months following at least one surgical procedure, with pain refractory to well-conducted conservative management under the guidance of a multidisciplinary pain clinic. Standard clinical practice at each site according to the French guidelines of the National Health Authority (HAS) for SCS selection and implantation facilitated SCS eligibility identification. Systematic evaluation of patients for ESTIMET study eligibility according to several inclusion criteria led to enrollment, before which, patients provided consent.

Study patients had a PSPS-T2 diagnosis with chronic back and leg pain characterized by neuropathic component (DN4 questionnaire, sensorimotor testing, clinical examination, pain characteristics, etc.); significant unilateral or bilateral radicular pain with mean intensity on a visual analogue scale (VAS) ≥ 50 mm (collected daily over five consecutive days) associated with a significant back pain component; and no further spine surgery indication.

Exclusion criteria included previous SCS treatment; subcutaneous or peripheral nerve stimulation; an intrathecal drug delivery system; experimental treatments; back surgery at the site related to the patient’s original back complaint requirement; and/or presenting a surgical, anesthetic or psychiatric contraindication to SCS treatment.

Patients who did not meet all of the inclusion criteria or who met at least one of the exclusion criteria were discontinued from the study.

### 2.4. Procedures

**Figure 1:**
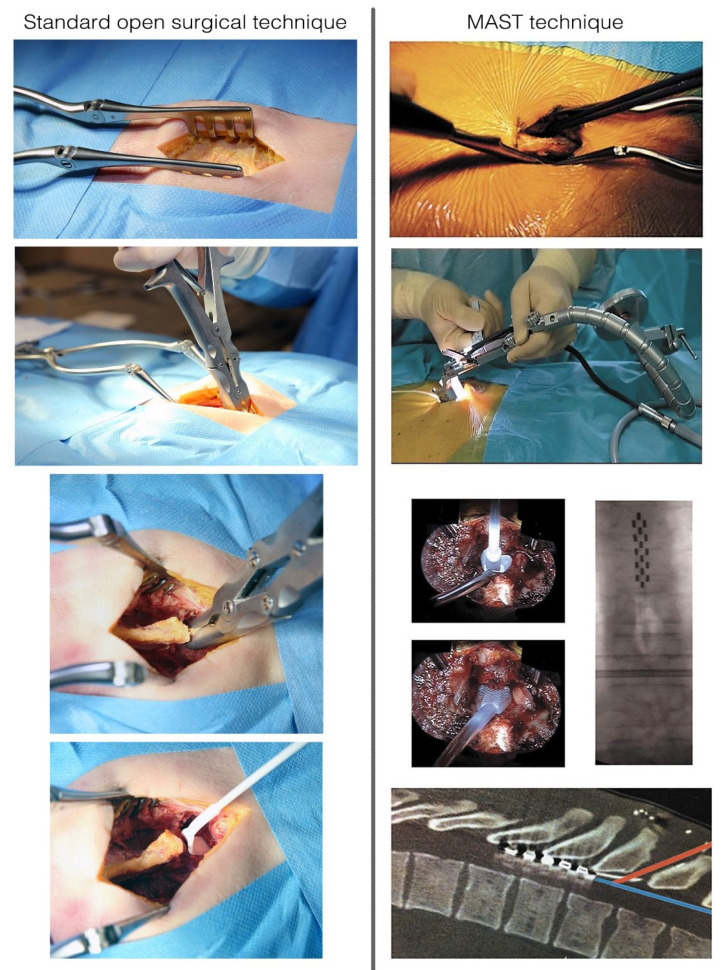
Conventional surgical approach (**Left**). The surgical approach consists in separating paraspinal fascia and muscles from the spinous process and ligaments. Once the spinous process and laminae have been exposed, removal of the supraspinous, the interspinous ligament and the ligamentum flavum is possible thanks to a small gouge or an arthrectomy pinch. Kerrison rongeurs can be used to remove a small portion of the inferior lamina of the upper vertebra to place the lead phantom and then the paddle lead in the epidural space. Aspects of the minimally invasive (MAST) procedure (**Right**). The surgical approach on one or both sides of the supraspinous process, with careful dissection of the paravertebral musculature. Full system set. Insertion of the phantom lead and implantation of the lead in the median position, verified by intraoperative X-ray. The aspects of lead implantation angle: an approach at the thoracic spine level is possible by using the minimally invasive technique. The bony removal can be minimized, and a shallow, safe angle of insertion achieved with a retractor system and illumination.

**Figure 2:**
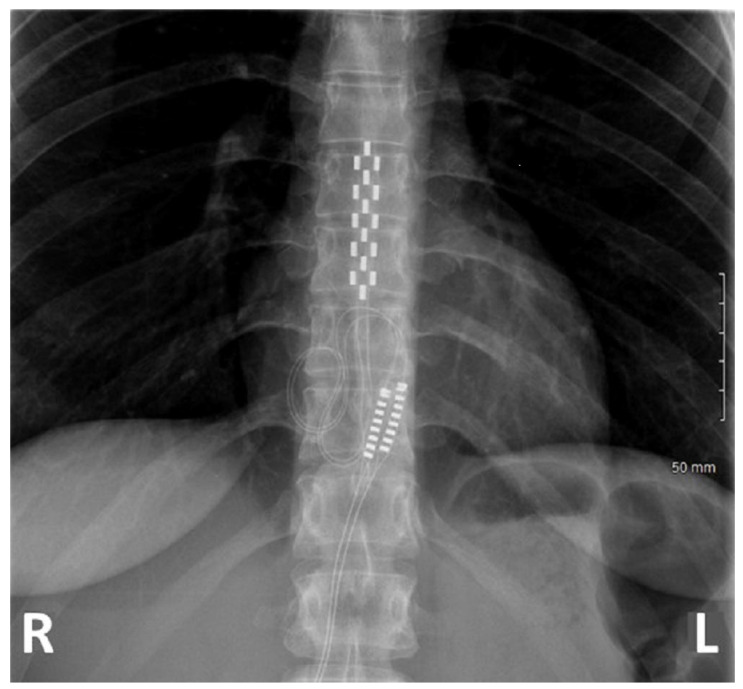
X-ray of a patient implanted at T8-T10 with a multicolumn lead.

All patients were implanted with a multicolumn SCS surgical lead (Specify 5-6-5, Medtronic, Minneapolis, MN 55432, USA). All neurosurgeons were considered experts in surgical SCS implantation. Additional implantation guidelines and two cadaver courses were used to homogenize implanting practices for both MIS and laminectomy. For both techniques, we performed a skin incision until visualization of the thoracic aponeuroses was achieved. The entry point of tissue resection was delineated by the selected interspinous space and surgical approach was performed both sides of the supraspinous process were surgically approached [38]. Laminectomy consisted of resecting spinous processes and laminae by an open approach using traditional retractors, as described in classical textbooks of neurosurgery [39], until a window adequate to access the epidural space and implant the multicolumn lead was created (Figure 1) [38]. MIS consisted of using a specific retractor dedicated to MAST (Quadrant, Medtronic, Minneapolis, MN 55432, USA), which was then carefully inserted into the inter-myo-laminar space at an angle ranging from 35° to 45° (Figure 1). After opening the retractor (30 mm), a cold light was attached to the retractor to ensure optimal visualization and access to the surgical field. The supraspinous and interspinous ligaments were resected, followed by the ligamentum flavum under optic magnification. Transverse (4 to 5 mm on each side) and craniocaudal direction (3 to 5 mm) dissections were performed. For both techniques, the lead was implanted in the median position and verified by X-ray (Figure 2). The lead was secured to the supraspinous ligament after the retractor system was withdrawn.

All patients were implanted in the prone position. The lead tip was positioned between T7 and T10, based on the level of the conus medullaris which was assessed by preoperative spinal magnetic resonance imaging. A radiographic assessment of lead position was conducted prior to wound closure in order to ensure anatomic midline placement, thoracic implantation level and lack of lead twisting. Finally, an impedance check ensured system integrity.

In all 11 centers, other than center X, patients were implanted under conventional general anesthesia. Leads were implanted over the spinal cord segments which were supposed to receive the dorsal root fibers corresponding to the painful segment of the back.

**Figure 3:**
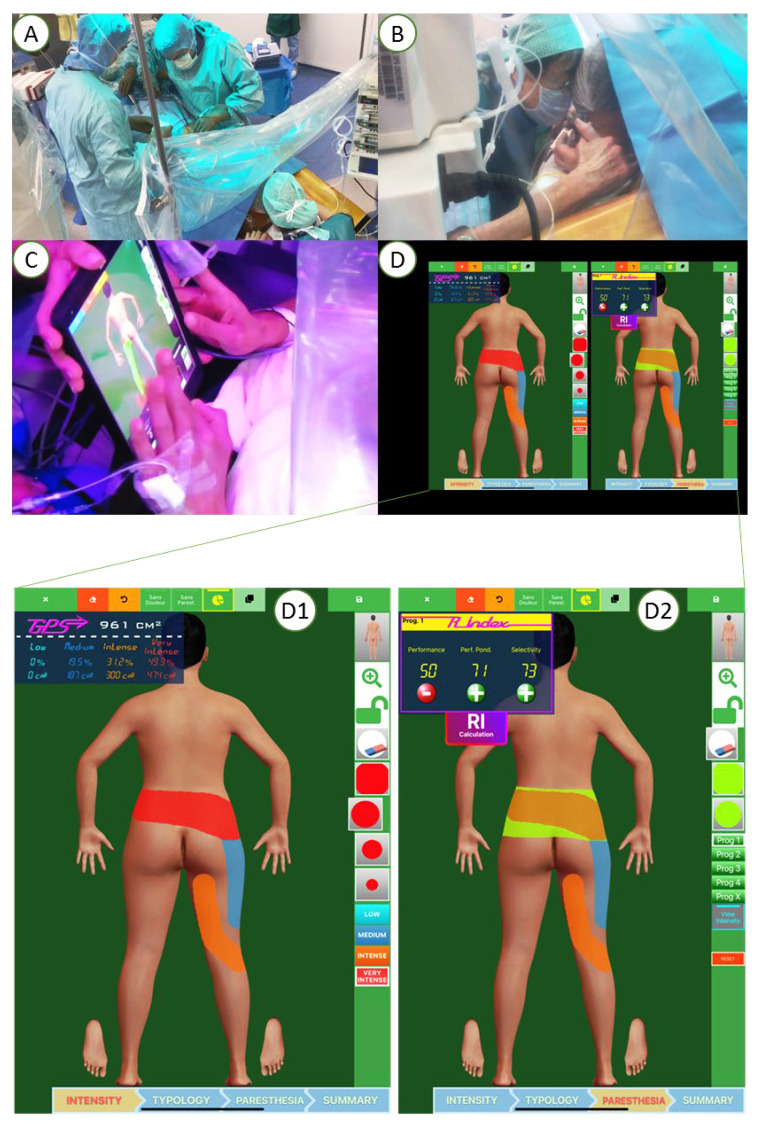
Surgical lead implantation (**A**) under awake surgery with intraoperative hypnosis (**B**) which enabled lead placement on the physiologic midline by intraoperative testing according to patient-reported optimal paresthesia coverage (**C**,**D**). Pain mapping software used to assess pain surface before implantation (**D1**) and pain coverage where the patient could draw different painful zones intraoperatively (**D2**).

In center X, patients were implanted under awake surgery by target-controlled intravenous anesthesia (TCIVA), which enabled lead placement on the physiologic midline by intraoperative testing according to patient-reported optimal paresthesia coverage. During TCIVA, the anesthesiologist administered a precise concentration of anesthetic agents (propofol and remifentanil), with the objective being to achieve the adequate brain concentration level. The doses required to achieve and maintain this target concentration were calculated and administered by a specific medical device that combined a calculation module, a pharmacokinetic model and a self-pulsing syringe. The main advantage of this type of anesthesia is to control in real time the depth of narcosis, and to, thereby, obtain a rapidly reversible loss/gain of consciousness of the patient. Another advantage of the TCIVA is the possibility of preserving spontaneous ventilation. In addition, the absence of diffusion of the anesthetic product in the spinal canal allows us to avoid a concentration gradient and consequently a neural block gradient, which distorts the electrode parameterization intraoperatively. Patients also had the option of receiving intraoperative hypnosis by a qualified hypnotherapist and/or a virtual reality device (Figure 3) [29]. During the lead implantation surgery, patients also had access to a pain mapping tool (Figure 3) [5,21,34,35,36,40,41]. The pain mapping software is a numerical tactile interface where the patient can draw different painful zones based on intensity represented by 4 different colors: red for very intense pain, orange for intense pain, dark blue for medium pain and light blue for low pain. This mapping software provides objective pain surface area and paresthesia coverage by converting pixels in cm² using a patented data processing system (Patent Applications N PCT/EP2014/067231, N PCT/FR 14/000 186 and N PCT/FR 14/000 187). The patients draw their pain surface on a body map, and also draw intraoperatively the paresthesia coverage following stimulation [35]. This allowed the implanter of patients under awake anesthesia to adjust the lead position intraoperatively until achieving optimal performance and selectivity.

Data were collected from patients at baseline, and at the 1-, 3-, 6- and 12-month follow-up visits.

### 2.5. Subgroup Analysis

Patients were allocated retrospectively to one of the three following sub-groups:

(1) The first sub-group includes patients having undergone optimized lead positioning through minimal invasive surgery under awake anesthesia (MISAA group) using TCIVA.
(2) The second group includes patients having undergone lead placement through minimal invasive surgery under general anesthesia (MISGA group).
(3) The third sub-group includes patients having undergone anatomic lead placement with laminectomy under general anesthesia (LGA group).

### 2.6. Study Outcomes

The pain mapping outcomes were considered as primary outcomes with the two following components:

- Paresthesia coverage was evaluated as the percentage of pain covered by paresthesia. This percentage was calculated as the surface of pain (in cm²) covered by paresthesia divided by the total pain surface.
- Selectivity was evaluated as the percentage of paresthesia covering pain (i.e., the surface of pain covered by paresthesia divided by the total paresthesia surface).

The following clinical outcomes were considered as secondary outcomes between baseline, 6- and 12-month follow-up visits:

- Global pain relief was evaluated by the percentage of global pain decrease and the percentage of patients with a 50% pain decrease.
- Back pain relief was evaluated by the percentage of back pain decrease.
- Leg pain relief was evaluated by the percentage of leg pain decrease.
- Functional improvement was evaluated by the percentage of decrease of the Oswestry disability questionnaire (ODI).
- Quality of life improvement was assessed using the absolute increase in EuroQoL-5 dimensions 3 level (EQ-5D-3L) index.
- Depression level was evaluated using the Montgomery–Asberg depression scale (MADRS).
- Holistic composite evaluation was used to provide a score representing the patient’s Global Health Score (GHS). This score includes pain intensity (VAS), functional disability (ODI), quality of life (EQ5D), depression (MADRS) and pain surface. The score was calculated based on a principal component analysis (PCA) by including these 5 variables [5,42]. The first principal component served as the global multidimensional score. The score was then scaled to 0 (worst global health) to 10 (best global health) for easier interpretations. The absolute difference between baseline and each follow-up was used to evaluate overall health improvement.
- Complication rates following the lead implantation were also reported and compared between the three groups.

### 2.7. Statistical Analysis

The statistical analyses were conducted using R software (Version 3.6.0, R Foundation for Statistical Computing, Vienna, Austria).

Baseline characteristics were described for each group using means (standard deviation) for quantitative variables or number (percentage) for qualitative variables. In order to verify the comparability of the three groups, their baseline characteristics were compared using a one-factor ANOVA test for continuous variables (or a Kruskal–Wallis test in case of non-normality) and a Chi-squared test (Fisher’s exact test in case of small numbers) for qualitative variables. Normality was verified using a Shapiro–Wilk test.

Continuous outcomes were compared between the three groups using either a one-factor ANOVA or a Kruskal–Wallis test depending on the normality of the outcome. Qualitative outcomes were compared using either a Chi-squared test or Fisher’s exact test.

In case baseline characteristics showed a significant difference between the three groups, these characteristics were then included in a multiple regression model as confounding variables when comparing the outcomes between the three groups.

When the one-factor ANOVA of an outcome showed a significant difference between the three groups, then multiple pairwise comparisons were conducted between each two groups to identify which groups were significantly different from the others. Since we conducted multiple comparisons, a Bonferroni correction was applied on the *p*-values to adjust for the false discovery rate.

*p*-values lower than 0.05 were considered significant, and all tests were two-tailed. No missing value imputation was conducted, and data were analyzed based on an available-case principle.

## 3. Results

### 3.1. Patient Characteristics

**Figure 4:**
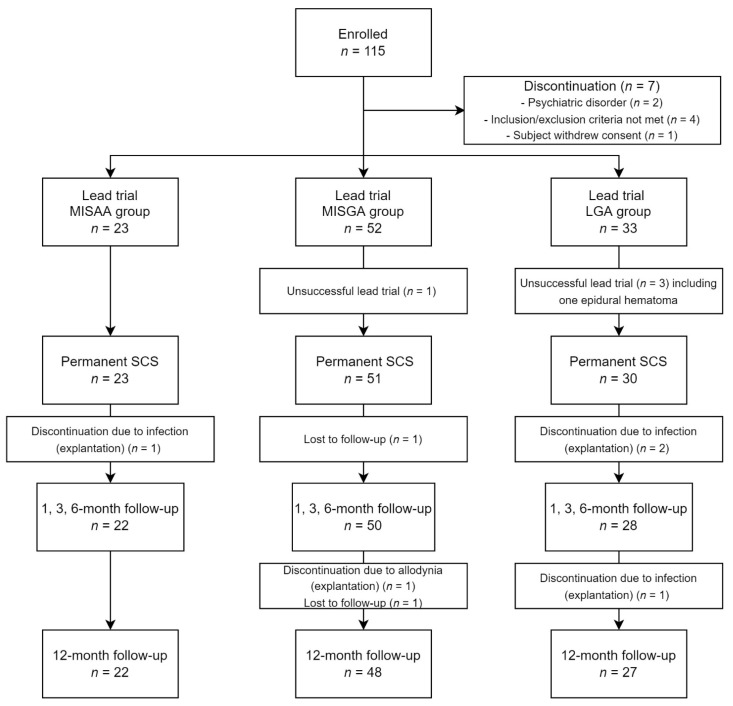
Study flow chart.

A total of 115 PSPS-T2 patients were included in the study. Two patients were excluded due to psychiatric disorders, and four patients were excluded for meeting an exclusion criterion. Among the remaining 109 patients, 1 patient withdrew his consent before lead implantation. A total of 108 patients underwent a multicolumn lead trial; 23 patients were implanted with a lead using the minimal invasive surgery awake anesthesia (MISAA group), while 52 patients were implanted by minimal invasive surgery under general anesthesia (MISGA group), and the remaining 33 patients were implanted by laminectomy under general anesthesia (LGA group). The final analyzed sample consisted of 22 patients in the MISAA group, 48 patients in the MISGA group and 27 patients in the LGA group (Figure 4).

Baseline characteristics of the study groups are presented in Table 1. The mean age of the three groups was 46.4 ± 7.6 years for the MISAA group, 50.7 ± 9.5 years for the LGA group and 46.2 ± 9.4 years for the MISGA group, without any significant difference between groups (*p* = 0.21). Gender distribution was not significantly different between groups (*p* = 0.62) with 39.1%, 50% and 51.5% of males in the MISAA, MISGA and LGA groups, respectively. The MADRS score was significantly higher for the MISAA (20.0 ± 9.8) and LGA (21.3 ± 11.8) than the MISGA (14.1 ± 9.7) group (*p* = 0.005). The number of previous spinal surgeries was significantly higher for the LGA and MISGA (median: 2, range: 1–5) groups in comparison with the MISAA group (median: 1, range: 1–3) (*p* = 0.026). The MADRS and number of previous spinal surgeries were therefore included in a regression model as confounding variables in order to compare the study outcomes between the three groups. The other baseline characteristics were not significantly different between groups (please see Table 1 for details).

Lead implantation parameters and IPG characteristics are presented in Table 2. We found a significant difference in lead lateralization (*p* = 0.003) and in permanent IPG type (i.e., rechargeable/non-rechargeable) (*p* = 0.0006) between groups.

Regarding lead lateralization for the MISAA group, 30.5% of the leads had a lateralization on the right in the upper part and on the midline in the lower part. For the LGA and MISGA groups the majority of leads were implanted at the midline in both the upper and the lower parts (60.6% of the leads for the LGA group and 55.8% for the MSIGA group). Regarding the type of IPG, the majority (69.6%) of IPGs were non-rechargeable for the MISAA group. Similarly, the majority (78.8%) of IPGs were non-rechargeable for the LGA group. For the MIGA group, on the other hand, the majority (53.8%) of IPGs were rechargeable.

The remaining device-related parameters were similar between the three groups (*p* > 0.05).

### 3.2. Comparison of Pain Mapping Outcomes between the Groups

The comparisons of paresthesia coverage, selectivity and pain surface at each follow-up visit are presented in Table 3.

**Figure 5:**
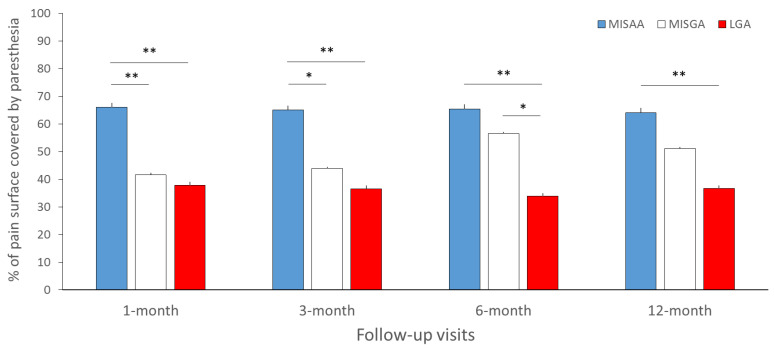
Mean paresthesia coverage (% of pain surface covered by paresthesia) for the MISAA (in blue), MISGA (in white) and LGA (in red) groups at the 1-, 3-, 6- and 12-month follow-up visits. MISAA: minimal invasive surgery under awake anesthesia; MISGA: minimal invasive surgery under general anesthesia; LGA: laminectomy under general anesthesia. * *p* < 0.05, ** *p* < 0.01, significant difference between groups.

Paresthesia coverage (% of pain covered by paresthesia) was significantly different between groups at the 1- (*p* = 0.01), 3- (*p* = 0.014), 6- (*p* = 0.003) and 12-month follow-up periods (*p* =0.03) (Figure 5). More specifically, at the 1- and 3-month follow-ups, the paresthesia coverage was greater in the MISAA group (66.0 ± 34.6%; 65.1 ± 33.7%) compared to the MISGA (41.6 ± 34.0%, *p* = 0.009; 43.8 ± 31.8%, *p* = 0.017) and LGA groups (37.8 ± 33.0%, *p* = 0.007; 36.5 ± 36.0%, *p* = 0.0096). In addition, paresthesia coverage was greater for the MISAA in comparison with LGA at the 6- (65.3 ± 38.2% vs. 33.8 ± 31.9%, *p* = 0.004) and 12-month (64.1 ± 36.7% vs. 36.6 ± 37.4%, *p* = 0.015) follow-ups, and greater for the MISGA group compared to the LGA group at the 6-month follow-up (MISGA: 56.5 ± 32.1% vs. LGA: 33.8 ± 31.9%, *p* = 0.008). No difference was found between paresthesia coverage of the MISAA and MISGA groups at the 6- (*p* = 0.15) and 12-month (*p* = 0.12) follow-ups, and between MISGA and LGA at the 12-month follow-up (*p* = 0.11).

Lead selectivity (% of paresthesia covering pain area adequately) was not significantly different between groups at the 1-month follow-up (*p* = 0.11), whereas a significant main effect of group was observed at the 3- (*p* = 0.011), 6- (*p* = 0.0008) and 12-month (*p* = 0.011) follow-ups. More specifically, at 3, 6 and 12 months, selectivity was significantly higher for the MISGA group (59.4 ± 33.4% at 3 months; 61.8 ± 33.6% at 6 months; 55.3 ± 36.0% at 12-months) compared to the MISAA group (30.8 ± 27.2%, *p* = 0.0026; 26.5 ± 23.7%, *p* = 0.002; 27.5 ± 22.7%, *p* = 0.008). In addition, selectivity was higher for the MISGA than LGA group at the 6- (61.8 ± 33.6% vs. 37.2 ± 36.6%, *p* = 0.024) and 12-month (55.3 ± 36.0% vs. 30.1 ± 34.9%, *p* = 0.024) follow-ups. No significant difference in selectivity was observed between MSIGA and LGA at the 3-month follow-up (*p* = 0.11), and between MISAA and LGA groups at the 3- (*p* = 0.44), 6- (*p* = 0.59) and 12-month follow-ups (*p* = 0.64).

The percentage of pain surface decrease was significantly different between groups at the 1- (*p* = 0.038) and 3-month (*p* = 0.019) follow-ups, but not at the 6- (*p* = 0.9) and 12-month (*p* = 0.42) follow-ups. More specifically, at 1 and 3 months, the percentage of pain surface decrease was higher for the MISAA (76.2 ± 30.5%; 68.5 ± 38.6%) than for the MISGA group (54.0 ± 38.1%, *p* = 0.012; 44.0 ± 37.8%, *p* = 0.008). No significant difference was found between the MISAA and LGA groups (*p* > 0.147), or between the MISGA and LGA groups (*p* > 0.146).

### 3.3. Comparison of Clinical Outcomes between Groups after 6 and 12 Months

Clinical outcomes between groups after 6 and 12 months are presented in Table 4.

#### 3.3.1. Overall Pain Relief

Overall VAS relief was significantly different between groups at 6 months (*p* = 0.047), but not at 12 months (*p* = 0.076). At 6 months, overall pain VAS relief was higher for the MISAA group (60.4% ± 31.9%) compared to the LGA group (35.4% ± 39.4%, *p* = 0.023), without any significant difference with the MISGA group (45.5% ± 32.8%, *p* = 0.076). No significant difference was found between the MISGA and the LGA groups (*p* = 0.26).

#### 3.3.2. Back Pain Relief

Back pain VAS relief was significantly different between groups at 6 months (*p* = 0.0037), but not at 12 months (*p* = 0.08). More specifically, at the 6-month follow-up, back pain VAS relief was significantly lower for the LGA group (18.9% ± 39.3%) compared to the MISAA (52.6% ± 35.6%, *p* = 0.0021) and MISGA (43.2% ± 34.1%, *p* = 0.0036) groups. No significant difference in back pain relief was observed between the MISAA and MISGA groups (*p* = 0.32) at 6 months.

#### 3.3.3. Leg Pain Relief

Leg pain VAS relief was significantly different between groups at the 6- (*p* = 0.016) and 12-month (*p* = 0.012) follow-ups, with significant higher leg pain VAS relief in the MISAA group (79.0% ± 22.3% at 6 months; 73.5 ± 26.5% at 12 months) in comparison with the MISGA (55.7% ± 34.3%, *p* = 0.0041; 52.9% ± 31.7%, *p* = 0.0051) and the LGA (54.2% ± 44.2%, *p* = 0.011; 46.4% ± 36.7%, *p* = 0.0061) groups. No significant difference was observed between LGA and MISGA groups at the 6- (*p* = 0.93) and 12-month (*p* = 0.41) follow-ups.

#### 3.3.4. Functional Capacity Improvement (ODI Score)

ODI score improvement was significantly different between groups at the 6- (*p* = 0.028) and 12-month (*p* = 0.033) follow-ups. ODI score improvement was significantly higher for the MISAA group (44.4 ± 32.5% at 6 months; 48.0 ± 28.3% at 12 months) than the MISGA group at the 6-month (26.8 ± 34.0%, *p* = 0.054) and 12-month (27.5 ± 36.5%, *p* = 0.019) follow-ups, and higher than the LGA group at the 12-month follow-up (27.5 ± 36.5%, *p* = 0.024). No significant differences of the ODI score improvement was observed between the MISAA group and the MISGA group at 6 months (*p* = 0.054), and the LGA group at 12 months (*p* = 0.11), or between the MISGA and LGA groups at 6 (*p* = 0.72) and 12 months (*p* = 0.54).

#### 3.3.5. Health-Related Quality of Life Improvement (EQ-5D-3L)

EQ-5D-3L score improvement was significantly different between groups at the 6- (*p* = 0.039) and 12-month (*p* = 0.015) follow-ups. EQ-5D-3L score improvement was significantly higher for the MISAA group (0.31 ± 0.19 at 6 months; 0.26 ± 0.16 at 12 months) than the MISGA group at 6 (0.14 ± 0.25, *p* = 0.0028) and 12 months (0.12 ± 0.27, *p* = 0.011), and for the LGA group (0.25 ± 0.22) than the MISGA group (0.12 ± 0.27, *p* = 0.035) at 12 months. No significant difference was found between the MISAA and LGA groups at 6 (*p* = 0.074) and 12 months (*p* = 0.96), or between MISGA and LGA at 6 months (*p* = 0.73).

#### 3.3.6. Depression Score Decrease (MADRS)

MADRS score decrease was significantly different between groups at 6 (*p* = 0.0003) and 12 months (*p* = 0.0005). MADRS score decrease was significantly higher for the MISAA than the MISGA group at 6 (12.3 ± 12.0 vs. 1.8 ± 7.2, *p* < 0.0001) and 12 months (12.2 ± 9.4 vs. 2.9 ± 9.9, *p* = 0.0002). MADRS score decrease was significantly higher for the MISAA than the LGA group at 6 (12.3 ± 12.0 vs. 3.5 ± 10.2, *p* = 0.009) but not at 12 months (12.2 ± 9.4 vs. 6.5 ± 10.5, *p* = 0.072). The MADRS score of the MISGA group was not significantly different from the score of the LGA group at 6 (*p* = 0.24) and 12 months (*p* = 0.27).

#### 3.3.7. Global Health Score Improvement (GHS)

The first component in the PCA representing the global health score explained 57% of the common variance in the VAS, ODI, EQ5D, MADRS and pain surface. The GHS score improved significantly following SCS implantation, rising from 2.1 ± 2.2 to 6.1 ± 3.5 at 6 months (*p* < 0.0001), and to 6.6 ± 3.3 at 12 months (*p* < 0.0001).

GHS improvement was significantly different between groups at the 6- (*p* = 0.022) and 12-month (*p* = 0.018) follow-ups. GHS improvement was significantly higher for the MISAA group than for the MISGA group at 6 (5.8 ± 2.9 vs. 3.5 ± 3.1, *p* = 0.006) and 12 months (6.0 ± 2.5 vs. 3.7 ± 3.3, *p* = 0.004), and then the LGA group at 6 months (3.9 ± 3.4, *p* = 0.028). No significant difference was found between the MISAA and LGA groups at 12 months (*p* = 0.21), or between MISGA and LGA at 6 months (*p* = 0.52) and 12 months (*p* = 0.17).

### 3.4. Safety Analysis

All in all, 65 adverse events were observed among the implanted 108 patients. The most frequent adverse event was postoperative pain following lead and IPG implantations (24/108 implanted patients). The rates of postoperative device-related pain were significantly different among the three groups (*p* = 0.00074), with at least one adverse event occurring in 11/23 (47.8%) patients in the MISAA group, 12/52 (23.1%) in the MISGA group and 1/28 (3.6%) in the LGA group.

The infection rates (11/108 patients) were significantly different between the groups (*p* = 0.00033) with 4/23 patients (17.4%) in the MISAA group, 0/52 (0%) patients in the MISGA group and 7/28 patients (25.0%) in the LGA group. Two patients in the LGA group experienced epidural hematoma, which required an emergency reoperation.

## 4. Discussions

By investigating SCS performance, where implantation was achieved by combining MIS with AA (MISAA group), our study showed that intraoperative testing performed under awake anesthesia to optimize lead placement led to greater performance on short- (1 and 3 months) and long-term (6 and 12 months) outcomes, in comparison with SCS surgical implantation performed under general anesthesia, wherever the lead was placed using MIS or open-surgical technique (MISGA and LGA groups). In addition, it appeared that greater SCS performance, assessed with an electronic interface, led to better clinical outcomes, since higher pain relief was obtained in the MISAA group for global and back pain at 6 months, and at 6 and 12 months for leg pain compared to MISGA and LGA groups.

### 4.1. Potential Added-Value of MIS to Allow Surgical SCS Placement under Awake Conditions

In this study, all patients were implanted with a multicolumn SCS surgical lead by either MIS or laminectomy [37]. Originally designed for spinal decompressions and instrumentations, MIS has been shown to reduce blood loss, muscle injury and postoperative pain, leading to improved recovery periods and shortened hospitalization [43,44,45,46,47,48]. More specifically related to SCS implantation, the MAST approach appears to facilitate visualization of the spinal canal and to optimize lead placement by facilitating pure transligamentar median lead placement [33]. While less invasive, this approach is considered as technically demanding and may result in higher incidence of complications [43,45,48]. Beyond the specific context of surgical SCS and in congruence with Richard North’s pivotal study comparing SCS vs. spine reoperation in PSPS-T2 patients [11], our results emphasize the fact that adequate pain coverage is an important pre-requisite to good patient outcome, when using tonic paraesthetic stimulation. In our innovative study, clinical outcomes were positively impacted by the MAST approach and awake anesthesia, which we were able to demonstrate with objective quantitative measurements using a dedicated software [5,21,34,35,36].

### 4.2. Combining MIS with TCIVA for Surgical SCS Implantation to Facilitate Patient Intraoperative Feedback

A previous prospective, multicenter study failed to report any differences of pain relief between patients implanted under awake or general anesthesia [49]. In contrast, our results showed that global, back and leg pain relief were greater for the MISAA group than for the MISGA and LGA groups at 6 months, and remained greater for leg pain relief at 12 months. In addition, pain surface decrease was greater for the MISAA (69–76%) than the MISGA (44–54%) and LGA (56–58%) groups at 1 and 3 months, while the difference diminished at 6 and 12 months, ranging from 50 to 64%, whatever the group. Similar results were found in favor of the MISAA group for clinical outcomes including functional capacity, quality of life, depression and, more meaningfully, for our MCRI composite score [5]. A multidimensional score, as recently recommended so as to ensure comprehensive assessment of complex pain [5,42,50], showed that patient health improvement was significantly greater in patients having undergone implantation under awake surgery and benefited from objective intraoperative assessment of SCS lead performance and selectivity. The literature has indeed emphasized the critical need of multidimensional assessment of pain [50] to more thoroughly explore psychological and functional dimensions that are liable to strongly influence the quality of life and global health of a given patient [4,5]. In a recent prospective study including 200 PSPS-T2 patients, we developed and proposed a new multidimensional clinical response composite index (MCRI) including pain intensity, pain surface, quality of life, psychological distress and functional disability based on a weighted mathematical algorithmic approach [5]. Having shown that the MCRI was able to better reflect the global health of the chronic pain patient than the standard metrics usually used in isolated form, via a digital interface this MCRI index has been introduced to assess the global health of chronic pain patients.

### 4.3. The Use of an Intraoperative Pain Mapping Tool Can Help to Optimize Lead Placement and Programming, Including Waveform Selection

We have noted the potential impact of an intraoperative tactile interface, not only in terms of high-fidelity assessment, but also as a catalyzer of patient intraoperative cooperation, leading to more precise information regarding spatial targeting and lead programming, thereby influencing the decision to change SCS lead anatomical placement or leave it as it is, according to patient SCS anatomy [51].

Ultimately, during the initial programming session and after spatial optimization, it appears possible to initiate intraoperative testing to orient the choice of possible waveforms [22,23] by negative selection. In fact, exclusion of further paraesthetic stimulation, if immediately uncomfortable sensations are reported by the patient (especially in case of preexisting allodynia), can influence the final lead placement and tune down the implanter’s intolerance to imprecision. Subsequently, if conventional stimulation modalities need to be directly waived from on-table testing, infra-paraesthetic stimulation modalities or waveform combinations will be tried from the beginning of the trial period, in order to maximize opportunities.

In light of these results, awake surgery appears to achieve greater outcomes than general anesthesia, especially by means of the MAST approach [32,33], which facilitates intraoperative testing with objective pain mapping tools [5,21,34,35,36].

By comparing 11 patients implanted in awake surgery and 9 patients implanted in general anesthesia, both with laminectomy approach, Falowski et al. [49] showed greater paresthesia coverage in general anesthesia than awake surgery at 6 weeks (83.5% vs. 46.6%). Considering our hypothesis that awake surgery is an interesting alternative means of improving electrode placement in cooperation with the patient, this result seems counterintuitive. First, the authors indicated that the patient provided verbal feedback regarding paresthesia coverage, while we used a quantitative mapping tool to objectively delineate SCS performance. Second, they concluded that electromyographic testing under general anesthesia is faster and leads to greater lead placement accuracy. On this subject, while Falowski et al. [49] published interesting results in general anesthesia and neuro-monitoring of such patients, the average procedure time exceeded 125 min, whereas the average operating time in our study reached a maximum of 60 min. In addition to considering that infection rates increase according to length surgical procedure, in a vulnerable population where infection risk oscillates between 2.5–14% [8,9,52,53,54], we believe that given the ineluctable plastic reorganization of sensitive neural networks, one should not exclusively consider motor responses of neuro-electro-monitoring on implanted patients. We assume that intraoperative live feedback remains precious in integrating sensitive changes of the central nervous system such as collateral allodynia, which could, because of relative hypoesthesia due to the initial nervous lesion, limit the clinical outcome, notwithstanding good lead performance or asymmetric response to SCS. Lastly, a motor response by intraoperative electromyography to select lead placement will not prevent jolting stimulation occurrence due to morphometrical spinal canal variability or dynamic impedance changes of the neural tissues at a certain level of stimulation; patient intraoperative feedback can.

However, one must remember that lead anatomical placement makes sense only if paresthesia/adequate coverage are needed and tonic conventional stimulation is used.

### 4.4. To What Extent Could Hypnosis Impact Patient Comfort during Awake Procedure?

It bears mentioning that awake surgery is clearly not considered as a gold standard for SCS implantation, and could be perceived as increasing stress/anxiety and discomfort [49,55]. However, previous studies have demonstrated that epidural anesthesia can achieve satisfactory analgesic level with limited discomfort during SCS implantation with laminectomy [56,57]. In view of addressing potential discomfort issues, hypnosis has been introduced intraoperatively and demonstrated its efficacy [29,58,59]. In this study, we assume that patient comfort unambiguously improved thanks to hypnosis, according to reported patient satisfaction and perception of the technique [29]. In contrast, we must admit that for some patients, a negative impact and perception could occur due to “a priori” beliefs and anxiogenic representation of hypnosis techniques, during preoperative patient education/preparation [60]. In fact, hypnosis can induce either a positive representation, markedly increasing therapeutical alliance, or else a negative perception, which will be unfavorable insofar as it aggravates patient anxiety [60] on entrance into the operating theatre for implantation. Our conviction is that intraoperative hypnosis could effectively optimize surgical implantation by adding comfort to a well-prepared patient and could decrease consumption of anesthetic drugs to make awake anesthesia more reversible [29]. It would also reinforce collaboration between the patient and the implanter, the objectives being to obtain more reliable feedback and optimal intraoperative mapping. More recently, the use of intraoperative virtual reality to facilitate hypnotic induction during surgical procedures has been introduced [61,62,63,64] and we have incorporated this approach in our daily routine. However, intraoperative hypnosis + MISAA + virtual reality requires significant human resources and would not be easy to standardize.

In this study, we were not able to demonstrate that intraoperative hypnosis led to decreased IV pharmacological anesthetic agent consumption, since the TCIVA model, which depends on the body mass index (BMI) and individual sensitivity thresholds, is not transposable from one patient to another. TCIVA is also technically demanding for the anesthesiologist, since placing a TCIVA patient in the prone position could lead to respiratory distress without it being possible to intubate in ventral decubitus. To avoid such complications, Vangeneugden [65] performed SCS implantation under spinal anesthesia, showing good results; however, as we aimed to transpose this approach to our practice, we observed a rostro-caudal gradient of diffusion of the anesthetic agents within the intrathecal and epidural space, which could modify patient perception intraoperatively and disturb paresthesia mapping [33]. Finally, this conclusion encouraged us to develop intraoperative hypnosis in combination with TCIVA so as to optimize surgical SCS implantation. Further studies including objective anesthetic drug consumption measurements will be required to more accurately assess its real benefits.

### 4.5. Study Limitations and Technical Considerations

Despite encouraging results, this study has some limitations. First, the retrospective nature of this ancillary study limits the potential of extrapolation, by presenting a certain level of asymmetry between the three different groups of patients (MISAA = 23, MISGA = 52, LGA = 33). In addition, MISAA technique was performed in only one center, which might induce some bias due to unobserved confounding factors. However, implanters were all considered as experts and the only difference was the implantation technique since the other aspects of the study were controlled/standardized. For further research, a prospective comparative design would be more appropriate to randomize surgical technique allocations, but at the price of potential ethical concerns and a lack of homogeneity between implanting centers.

*Technical considerations*. While SCS implantation under awake surgery showed encouraging results, this approach raises potential concerns and recommendations. Firstly, implanting an SCS multicolumn surgical lead while the patient is awake is technically demanding and should be reserved to experienced teams of anesthesiologists and spine surgeons.

Reported adverse events were more frequent in awake group (6/11: chest wall pain, numbness/weakness, loss of stimulation and unpleasant stimulation) than in the general anesthesia group (2/19: lead migration and persistent pain due to exacerbation of underlying back pain) [49]. Similarly, higher rates of adverse events with worse postoperative device-related pain were observed in the MISAA (11/23) group than in the MISGA (12/52) and LGA (1/28) groups. This result seems quite coherent, considering that compared to MISGA and LGA patients, MISAA patients were less under postoperative influence of anesthetic agents. Questioning the patient retrospectively, after the surgical procedure, might have represented a subjective bias and should be carefully interpreted. In addition, infection rates were lower for patients having undergone the MAST approach (4/23 for the MISAA group and 0/52 in the MISGA group = 4/75 for MIS) in comparison with patients having undergone an open procedure (7/28 for the LGA group). It is safe to assume that the MAST approach could reduce infectious risk, as has been demonstrated for minimal invasive spinal surgery [32,33].

All in all, this study, despite its limitations, highlights the added value of combining intraoperative optimized anesthetic conditions and objective assessment in view of optimizing lead placement.

### 4.6. Does Optimized Lead Positioning Matter in 2022?

As theoretical evidence needs to be transposable to daily practice, this study raises further questions. Indeed, it is far from sure that our conclusion regarding SCS surgical implantation would fit with percutaneous lead implantation practice. Moreover, in an era of multiplied adjustments with temporal resolution of the signal [66], the privileging of spatial adjustments over temporal adjustments remains contestable.

In fact, different stimulation modalities such as burst, high frequency and high density, are currently used to optimize temporal targeting [18,20,21,22,41], and can now be combined according to patient preference [23]. Other approaches included algorithmic optimization of stimulation frequency and pulse width based on the previously preferred parameters by a given patient [66]. This is achieved by predicting a new and untested set of parameters which would achieve an optimal preference using the Bayesian preference learning statistical approach.

A better option would probably consist of combining rather than opposing these two strategies [8]. Recent technological innovation offers new opportunities to shape and optimize the delivered electrical field by means of Multiple Independent Current Control (MICC^TM^) technology [21,28] or closed-loop technology [53]. Our vision is that when implanting SCS, neural spatial targeting, is of crucial importance, not only as it catalyzes spatial targeting by temporal resolution adjustments, but also in terms of electrical consumption, as it optimizes SCS lead implantation close to the target. One trend in our community consists in recommending specific vertebral levels of lead placement to capture specific dermatomes with a standardized approach, even under general anesthesia, which is opposite to the concept described in this study. In a prospective cohort of 76 implanted patients, it was shown that optimized lead positioning can make a difference, when two consecutive patients enrolled in a prospective study did not show the same projection of the conus medullaris, which varied between T11 and L2 vertebral levels [51]. Conceptually, SCS goal is aimed at establishing a dialogue between electronics and neural structures, which would be organized according not to vertebral, but rather to myelomeric distribution. To make it clear, rather than recommending implantation of a lead at the T9-T10 vertebral level to capture a selected dermatome, we should recommend implantation at +5 myelomere above the conus medullaris, for which the anatomical projection is eminently variable [51]. This concept, if adopted, would define a “true anatomical placement”. Adding another level of granularity to anatomical somatotopic distribution, electrophysiological organization of the neural tissues, especially in case of neural plasticity secondary to a nerve injury, which defines neuropathic pain [67], could possibly map the neural fibers through live feedback [40], thereby confirming that technical placement has been optimized. With this in mind, the added value of intraoperative objective mapping testing appears to play a central role. Combined MIS+TCIVA offers this opportunity.

### 4.7. The Use of Intraoperative Assessment by Pain Mapping Tool Combined with Awake and MIS Surgical SCS Implantation As a Surrogate for Lead-Trial Phase

Lead trial performed before any permanent device implantation, following the international recommendation [39,68,69,70], is intended to determine the potential added value offered by SCS during period of trial (>5 days) by identifying positive SCS responders [20,21] and to potentially optimize neural structure spatial targeting. However, we must note some opposing arguments before performing a trial phase. First, we must admit that increasing the number of implantation acts increases complication rates and the duration (over 14 days of trialing) increases infection rates [54]. Second, the use of unidimensional pain intensity to determine SCS success/effectiveness can no longer be considered as the gold standard of pain assessment insofar as pain includes multidimensional components such as quality of life, psychological distress and functional disability [4,5,42]. Third, despite the current regulatory requirement for a trial period, recent studies have failed to evidence the added value of a trial period on responder rates compared to implantation without trial phase [71] or with machine learning algorithm prediction [72]. Reinforcing previous arguments, awake surgery combined with intraoperative testing pain mapping assessment (surface area related to pain intensity and paresthesia coverage) could be considered as a valuable approach to optimize lead placement, suggesting permanent one-stage implantation.

## 5. Conclusions

In this study, it appeared that chronic refractory pain PSPS-T2 patients implanted with SCS could substantially benefit from a combination of minimal invasive surgery, intraoperative quantitative pain and paresthesia mapping, and intraoperative hypnosis in view of optimizing SCS lead placement. However, this combined strategy is technically demanding and should be reserved to dedicated centers.

Ultimately, intraoperative clinical assessment of SCS leading to technical performance and selectivity presents a clear opportunity to compare different techniques, different SCS programs and to guide lead choice and lead placement, independently from potential industry influence and based on objective and reliable comparative measurements of spatial targeting and temporal resolution optimization of the signal. This opportunity would require intraoperative patient feedback facilitated by the association of intraoperative techniques, objective assessment tools, hypnosis support and virtual reality developments. In an era of currently debated “No-Trial” [71,72], perspectives in favor of SCS direct implantation, designed to optimize technical aspects of SCS implantation, could help to delineate a crucial piece of this giant puzzle.

## Figures and Tables

**Table 1:** Baseline characteristic comparisons between the MISAA, MISGA and LGA groups.

KERRYPNX	MISAA Group *n* = 23	MISGA Group *n* = 52	LGA Group *n* = 33	*p*-Value of the Difference
Age (years)	46.4 ± 7.6	46.2 ± 9.4	50.7 ± 9.5	0.21
Sex male/female *n* (%)	9/14 (39.1%)	26/26 (50%)	17/16 (51.5%)	0.62
BMI (kg/m²)	26.7 ± 4.3	26.8 ± 4.7	28.0 ± 6.3	0.72
Pain duration (years)	11.7 ± 9.2	11.3 ± 9.7	12.8 ± 12.5	0.8
Global VAS (mm)	71.5 ± 13.3	76.0 ± 11.5	75.7 ± 14.2	0.33
Back pain VAS (mm)	71.1 ± 18.1	74.1 ± 16.9	75.9 ± 14.8	0.62
Leg pain VAS (mm)	75.7 ± 9.0	78.7 ± 11.1	73.9 ± 15.1	0.20
Pain surface in cm² (median (IQR))	522.7 (921.6)	807 (969.5)	503.4 (924.6)	0.12
Patients with predominant back pain back/leg *n* (%)	9/14 (39.1%)	15/37 (28.8%)	15/18 (45.5%)	0.28
Patients with a neuropathic component (%)				
			
Back pain *n* (%)	13/10 (56.5%)	29/23 (55.8%)	18/15 (56.3%)	0.99
Leg pain *n* (%)	22/1 (95.7%)	52/0 (100%)	33/0 (100%)	0.21
Number of previous spinal surgeries (median (min–max))	1 (1–3)	2 (1–5)	2 (1–5)	0.026
MADRS depression score	20.0 ± 9.8	14.1 ± 9.7	21.3 ± 11.8	0.005
BAS anxiety score	19.5 ± 8.2	17.7 ± 8.1	18.3 ± 6.5	0.57

BAS: brief anxiety scale; IQR: interquartile range; MADRS: Montgomery–Asberg depression rating scale; MISAA: minimal invasive surgery under awake anesthesia; MISGA: minimal invasive surgery under general anesthesia; LGA: laminectomy under general anesthesia; VAS: visual analogic scale.

**Table 2:** Lead implantation parameters and IPG characteristics for each group.

	MISAA Group *n* = 23	MISGA Group *n* = 52	LGA Group *n* = 33	*p*-Value of the Difference
Vertebral level projection of the conus medullaris				0.12
		
T11-T12	0 (0%)	5 (9.6%)	0 (0%)	
T12-L1	15 (65.2%)	28 (53.8%)	25 (75.8%)	
L1-L2	8 (34.8%)	16 (30.8%)	7 (21.2%)	
L2-L3	0 (0%)	0 (0%)	1 (3.0%)	
Unknown	0 (0%)	3 (5.8%)	0 (0%)	
Lead lateralization (Upper/lower) (*n*, %)				0.003
		
Right/Right	2 (8.7%)	9 (17.3%)	1 (3.0%)	
Right/Left	0 (0%)	0 (0%)	1 (3.0%)	
Right/Midline	7 (30.5%)	3 (5.8%)	6 (18.2%)	
Left/Right	0 (0%)	0 (0%)	0 (0%)	
Left/Left	0 (0%)	5 (9.6%)	1 (3.0%)	
Left/Midline	5 (21.7%)	3 (5.8%)	1 (3.0%)	
Midline/Right	1 (4.3%)	0 (0%)	1 (3.0%)	
Midline/Left	3 (13.0%)	3 (5.8%)	0 (0%)	
Midline/Midline	5 (21.7%)	29 (55.8%)	20 (60.6%)	
Unknown	0 (0%)	0 (0%)	2 (6.1%)	
Vertebral level projection of the central contact “number 8” of the 5-6-5 lead.				0.32
		
		
T11	1 (4.3%)	1 (1.9%)	0 (0%)	
T10	2 (8.7%)	8 (15.4%)	5 (15.2%)	
T9	16 (69.6%)	22 (42.3%)	14 (42.4%)	
T8	4 (17.4%)	15 (28.8%)	9 (27.3%)	
T7	0 (0%)	6 (11.5%)	1 (3%)	
T6	0 (0%)	0 (0%)	1 (3%)	
Unknown	0 (0%)	0 (0%)	3 (9.1%)	
IPG type				0.0006
Non rechargeable	16 (69.6%)	23 (44.2%)	26 (78.8%)	
Rechargeable	7 (30.4%)	28 (53.8%)	4 (12.1%)	
Not implanted permanently	0 (0%)	1 (1.9%)	3 (9.1%)	

IPG: internal pulse generator; MISAA: minimal invasive surgery under awake anesthesia; MISGA: minimal invasive surgery under general anesthesia; LGA: laminectomy under general anesthesia.

**Table 3:** Comparison of the pain mapping outcomes among the MISAA, MISGA and LGA groups at the 1-, 3-, 6- and 12-month follow-ups.

Follow-Up Visits	MISAA Group	MISGA Group	LGA Group	Adjusted *p*-Value *
1-month	*n* = 22	*n* = 50	*n* = 28	
Paresthesia coverage (%) Selectivity (%) % of pain surface decrease	66.0% ± 34.6% ^a^ 31.2% ± 28.0% 76.2% ± 30.5% ^a^	41.6% ± 34.0% ^b^ 53.6% ± 38.3% 54.0% ± 38.1% ^b^	37.8% ± 33.0% ^b^ 41.3% ± 37.8% 56.3% ± 41.4% ^a,b^	0.010 0.11 0.038
3-month	*n* = 22	*n* = 50	*n* = 28	
Paresthesia coverage (%) Selectivity (%) % of pain surface decrease	65.1% ± 33.7% ^a^ 30.8% ± 27.2% ^a^ 68.5% ± 38.6% ^a^	43.8% ± 31.8% ^b^ 59.4% ± 33.4% ^b^ 44.0% ± 37.8% ^b^	36.5% ± 36.0% ^b^ 42.4% ± 37.1% ^a,b^ 57.8% ± 34.6% ^a,b^	0.014 0.011 0.019
6-month	*n* = 22	*n* = 50	*n* = 28	
Paresthesia coverage (%) Selectivity (%) % of pain surface decrease	65.3% ± 38.2% ^a^ 26.5% ± 23.7% ^a^ 50.5% ± 44.4%	56.5% ± 32.1% ^a^ 61.8% ± 33.6% ^b^ 53.1% ± 41.7%	33.8% ± 31.9% ^b^ 37.2% ± 36.6% ^a^ 50.3% ± 38.4%	0.0033 0.0008 0.9
12-month	*n* = 22	*n* = 48	*n* = 27	
Paresthesia coverage (%) Selectivity (%) % of pain surface decrease	64.1% ± 36.7% ^a^ 27.5% ± 22.7% ^a^ 64.6% ± 40.3%	51.1% ± 34.5% ^a,b^ 55.3% ± 36.0% ^b^ 61.3% ± 34.8%	36.6% ± 37.4% ^b^ 30.1% ± 34.9% ^a^ 51.6% ± 42.4%	0.032 0.011 0.42

* *p*-values were adjusted for the differences among the groups and for multiple comparisons. Values with a different exponent were significantly different in the post-hoc pairwise analysis. MISAA: minimal invasive surgery under awake anesthesia; MISGA: minimal invasive surgery under general anesthesia; LGA: laminectomy under general anesthesia. ^a,b^ Values with a different exponent were significantly different in the post-hoc pairwise analysis.

**Table 4:** Comparison of the clinical outcomes between the AG and TCI groups at the 6- and 12-month follow-ups.

Clinical Outcomes at the 6-Month Follow-Up	MISAA Group *n* = 22	MISGA Group *n* = 50	LGA Group *n* = 28	Adjusted *p*-Value *
% of decrease in global VAS % of decrease in back VAS % of decrease in leg VAS % of decrease in ODI Absolute increase in EQ-5D-3L Absolute decrease in MADRS	60.4% ± 31.9% ^a^ 52.6% ± 35.6% ^a^ 79.0% ± 22.3% ^a^ 44.4% ± 32.5% ^a^ 0.31 ± 0.19 ^a^ 12.3 ± 12.0 ^a^	45.5% ± 32.8% ^a,b^ 43.2% ± 34.1% ^a,b^ 55.7% ± 34.3% ^b,a^ 26.8% ± 34.0% ^a,b^ 0.14 ± 0.25 ^a,b^ 1.8 ± 7.2 ^b,a^	35.4% ± 39.4% ^b^ 18.9% ± 39.3% ^b^ 54.2% ± 44.2% ^b^ 22.3% ± 25.4% ^b^ 0.17 ± 0.26 ^b^ 3.5 ± 10.2 ^a^	0.047 0.0037 0.016 0.028 0.039 0.0003
Clinical outcomes at the 12-month follow-up	MISAA group *n* = 22	MISGA group *n* = 48	LGA group *n* = 27	Adjusted *p*-value *
% of decrease in global VAS % of decrease in back VAS % of decrease in leg VAS % of decrease in ODI Absolute increase in EQ-5D-3L Absolute decrease in MADRS	59.0% ± 31.7% ^a^ 55.9% ± 38.0% ^a^ 73.5% ± 26.5% ^a^ 48.0% ± 28.3% ^a^ 0.26 ± 0.16 ^a^ 12.2 ± 9.4 ^a^	44.0% ± 36.2% ^a^ 42.7% ± 39.0% ^a^ 52.9% ± 31.7% ^b^ 27.5% ± 36.5% ^a,b^ 0.12 ± 0.27 ^b^ 2.9 ± 9.9 ^b^	35.9% ± 38.1% ^a^ 27.7% ± 44.0% ^a^ 46.4% ± 36.7% ^b^ 31.4% ± 28.8% ^b^ 0.25 ± 0.22 ^a^ 6.5 ± 10.5 ^a^	0.076 0.080 0.012 0.033 0.015 0.0005

VAS: visual analogic scale; ODI: Oswestry disability index; EQ-5D-3L: EuroQol-5 dimensions. * *p*-values were adjusted for the differences between the groups in MADRS and number of surgeries and for multiple comparisons. ^a,b^ Values with a different exponent were significantly different in the post-hoc pairwise analysis.

## Data Availability

Not applicable.
